# The Influence of Operations upon the Prolongation of Life and Permanent Recovery in Carcinoma of the Breast

**Published:** 1882-05

**Authors:** 


### THE INFLUENCE OF OPERATIONS UPON THE PROLONGATION OF LIFE AND PERMANENT RECOVERY IN CARCINOMA OF THE BREAST.

Dr. S. W. Gross, of Philadelphia, read a paper before the New York Academy of Medicine, on January 19, 1882, of which a full report, with the discussion thereon, is given in the *Medical Record* of January 28th.

After analyzing several collections of cases, Dr. Gross reached the conclusion that operation prevented implication of internal organs in 32.30 out of every one hundred cases.

Again, life may be prolonged and permanent cure effected by surgical intervention. Extirpation adds one year to life. Special attention was directed to Volkmann's statement that the result might be regarded as final if the patient survives over *three* years after the *last* operation. The author then presented an analysis of 524 cases, in which 1 in 9.19 fulfilled these requirements. Subjecting the 524 cases to Paget's severe test—that the patient should live more than ten years from the beginning of the disease, or that the disease should be stationary—he found that 1 in 5.7 fulfilled these requirements. The average duration after operation in all these cases, was from seven to ten months. An analysis of the 57 cases cured was then given, and the conclusion reached that recurrent tumors should be freely extirpated as soon as they appear.

The absence of glandular implication does not afford absolute guarantee that secondary deposis are not in the viscera.

Dr. Gross makes it a rule to amputate the entire mamma, search for any outlying nodules, dissect away the fascia overlying the pectoral muscle, open the axilla, and remove any glands which have escaped observation previous to interference. Heretofore, one cure out of every nine and one fifth cases has been the most expected from early radical measures ; but there was reason to believe that the ratio of cure would be increased. Partial operations should be *discarded,* for they are more fatal than removal of the entire breast, and they hold out but little prospect of permanent recovery. He believed that in the future the mortality from radical procedures would not reach ten per cent.

The conclusions reached were substantially as follows :

1. That surgical intervention in carcinoma of the breast tends to retard the progress of the disease by preventing local dissemination, implication of associated lymphatic glands, and the development of visceral tumors.

2. That local reproductions do not militate against permanent recovery, provided they are thoroughly and early excised, as soon as they appear ; and that lymphatic involvement does not forbid operation, since, in fact, glands were removed in more than one third of the examples of final cure.

3. That the subjects are almost without exception saved from local reproduction, if *three* years have elapsed after the last operation.

4. That the risk from operations is outweighed by benefits which accrue from them, since they not only add twelve months to the life of the patient, but also cure one half as many patients as they destroy.

5. That all carcinomas of the breast—if there is no evidence of metastatic tumors, and if thorough removal is practicable—should be dealt with as early as possible, by amputating the entire mamma, integument and all, dissecting away all the subjacent fascia, opening the axilla, with the view to exploration and removal of all the glands not palpable prior to interference.

In the discussion which followed the reading of the paper, the conclusions of Dr. Gross were generally acquiesced in by Drs. Geo. A. Peters, Weir, A. C. Post, Parker, Leale, Flint, Sayre, Hamilton, Barker and Satterthwaite. The latter, upon invitation, occupied the attention of the Academy chiefly with some statistics derived from the study of carcinoma in New York. Before presenting these facts, he stated that he personally felt that the profession owed a great debt to Dr. Gross for the careful and laborious study he had given to breast-tumors, and for the vigorous effort he had made to show that a certain definite clinical picture was associated with equally definite microscopic appearances.

The attainment of this result should be of great importance to the surgeon, as he is now able to recognize the special variety of disease, and make it tally with the advanced standard of pathological terminology, while he has at the same time an additional satisfaction in being able to give a certain mathematical precision to his prognosis. Indeed, the surgeon will now rarely make a mistake, if he notes the time of life at which the disease commenced, its duration, rate of growth, external appearances, feel, conditions of the nipple and of the adjacent glands.

Dr. Satterthwaite observed that in his experience we had only three varieties of carcinoma to deal with in the breast—scirrhus in its various forms, colloid and encephaloid. Of the former he would not speak, because its characters were so well known that a description would be superfluous. It was well enough, however, to allude to encephaloid, because it was frequently mistaken for sarcoma. The former, occurring in about three per cent of his cases, was surpassed in this regard by sarcoma, which occurred in four per cent. Encephaloid is a disease that belongs to a more advanced period of life than scirrhus, the age of fifty to fifty-one representing the date of its average appearance. It is apt to be a rapidly forming, often bulky tumor, which is associated with much pain, and may or may not implicate the nipple and neighboring glands. The surface of the tumor is usually soft, often semi-fluctuating, is apt to break down early and give the appearances that attend active inflammation. Its surface also is apt to be lobulated, and the growth will, if sufficiently advanced, adhere to the underlying tissues. Though this form of growth is very naturally associated in one's mind with a rapid and excessively malignant course, it was but proper to say that in two cases—the only ones of encephaloid of which he had extensive notes—one survived the inception of the disease five years, and the other eight or nine. The latter is now a private nurse in St. Luke's Hospital and is apparently in perfect health, having had no signs of recurrence since the first and only operation. The growth was removed four months after it was first detected.

Sarcomas are met with at an earlier period of life than encephaloid, attain a great bulk in a short time, but are thought to have a more chronic course on the whole, as they recur after considerable intervals. In them the nipples are occasionally retracted and there is implication of adjacent glands, while often the skin becomes involved, and later breaks down, allowing the new growth to protrude, forming, as in encephaloid, a "fungus hæmatodes." Such tumors are not, however, adherent to underlying parts. The surface is slightly lobulated, but smooth. On cutting open such a tumor with an ordinary knife, it will be seen to have a capsule, while encephaloid does not. The microscopic appearances are those of round or spindle-celled sarcoma. In addition, cysts are quite common, but both in sarcoma and in encephaloid there may be discharge from the nipple.

Colloid carcinoma occurs in about two per cent of all breast-neoplasms. It appears on an average at an earlier period than scirrhus, and is essentially chronic in its development. The surgeon is quite apt to mistake this variety for scirrhus, because the physical characters before operation simulate those of the former disease very closely. After removal, however, when it has been opened by the knife, the peculiar gelatinoid material exudes or can be expressed, and then the diagnosis of colloid is easily made. Of two cases in Dr. Satterthwaite's experience, where pretty full histories have been obtained, the patients were well at last accounts—one at the end of two and the other at the end of six years after the origin of the disease. As to its danger of being confounded with myxoma, Dr. Satterthwaite was not prepared to say anything, as he had never met this form of tumor in the breast. Colloid was the most chronic of all these varieties.

These statistics, and others he was able to furnish, had been collected from records of eighty-six cases that had come into his possession. All of the cases had been restudied during the summer of the past year, and in all thirty-six cases were sufficiently complete to furnish material for statistical work. He enumerated some of the chief points that he had elicited. Carcinoma (all varieties) never appeared before twenty-eight or later than seventy. In ninety-seven per cent it attacked the female breast, and in three per cent the male. Usually it was the right breast. The most frequently assigned cause was some form of traumatism, including under that term such accidents as a blow, the chafing of corsets, friction of the breasts against a washing-board, mammary abscess, injury by child during lactation. Heredity was a factor that was assigned in a much smaller number of cases, say in one sixth. In about seventy-three per cent the previous health was said to have been good. Recurrences took place, on the average, about the tenth month after the operation ; but, if the patient had no recurrence during three years, a cure might be predicted. This conclusion was singularly precise in its agreement with the statements of Dr. Gross. The last recurrence occurred exactly at the thirty-sixth month.

As to the matter of cure, he was glad that he was able to say of metropolitan practice that it just about reached the high standard of excellence set by Dr. Gross. Taking thirty-one cases in which this matter could be properly studied, and accepting the fact that no recurrence had taken place for three years after operation, a cure might be claimed. Dr. Satterthwaite was able to say that three cases were definitely cured. This was a percentage of 9.68 per cent. In these cases the period of immunity lasted from six to ten years. On the other hand, a check was given to the disease in two cases, the duration of life after the origin of the disease being respectively about five and twenty-four years. He was also able to say that the dangers of the operations in this vicinity were trivial as compared with the statistics of Dr. Gross. The actual mortality, carrying the computation to the eighth day after the operation, was less than three per cent.

In conclusion, he had a word to say upon the comparative advantage of early and late operations. This was a topic, he was free to confess, he approached with hesitation, because the statistics, both on this and on another occasion when he had studied them, gave little encouragement to the surgeon who advocates early operation. And yet he felt impressed with the idea that early operations, and in recurrence, frequent operations offer the best chances of securing immunity or retardation. To be brief, there was a disturbing element in the computation, and it was the fact that benign tumors became unavoidably confused with the malignant. That he believed to be the case in the examples of cure or retardation already alluded to. Thus, in the cases of cure while two were operated on early—one at the end of four months and the other at the end of six—the third went five years without operation ; and in the retarded cases, one was not treated surgically until two, and the other until eighteen years had passed after trouble was first noticed in the breast. Could it not be, and was it not probable, from the history, that these instances of late operation were in cases where the growths were at first benign ?

Perhaps, at a later period in the study of tumors, when the number of carefully recorded cases is much larger, such as these might be thrown out of the calculation, and we should be able to decide this very important question.

